# Behavioral characterization of a CRISPR-generated TRPA1 knockout rat in models of pain, itch, and asthma

**DOI:** 10.1038/s41598-020-57936-5

**Published:** 2020-01-22

**Authors:** Rebecca M. Reese, Michelle Dourado, Keith Anderson, Søren Warming, Kimberly L. Stark, Alessia Balestrini, Eric Suto, Wyne Lee, Lorena Riol-Blanco, Shannon D. Shields, David H. Hackos

**Affiliations:** 1 Department of Neuroscience, Genentech, 1 DNA Way, South San Francisco, CA 94080 USA; 2 Department of Immunology, Genentech, 1 DNA Way, South San Francisco, CA 94080 USA; 3 Department of Molecular Biology, Genentech, 1 DNA Way, South San Francisco, CA 94080 USA

**Keywords:** Ion channels in the nervous system, Chronic pain

## Abstract

The transient receptor potential (TRP) superfamily of ion channels has garnered significant attention by the pharmaceutical industry. In particular, TRP channels showing high levels of expression in sensory neurons such as TRPV1, TRPA1, and TRPM8, have been considered as targets for indications where sensory neurons play a fundamental role, such as pain, itch, and asthma. Modeling these indications in rodents is challenging, especially in mice. The rat is the preferred species for pharmacological studies in pain, itch, and asthma, but until recently, genetic manipulation of the rat has been technically challenging. Here, using CRISPR technology, we have generated a TRPA1 KO rat to enable more sophisticated modeling of pain, itch, and asthma. We present a detailed phenotyping of the TRPA1 KO rat in models of pain, itch, and asthma that have previously only been investigated in the mouse. With the exception of nociception induced by direct TRPA1 activation, we have found that the TRPA1 KO rat shows apparently normal behavioral responses in multiple models of pain and itch. Immune cell infiltration into the lung in the rat OVA model of asthma, on the other hand, appears to be dependent on TRPA1, similar to was has been observed in TRPA1 KO mice. Our hope is that the TRPA1 KO rat will become a useful tool in further studies of TRPA1 as a drug target.

## Introduction

Transient receptor potential family member ankyrin 1 (TRPA1) is an ion channel that is expressed in sensory neurons of the peripheral nervous system^1,2^. This channel is unusual in that it can be activated by a wide variety of environmental irritants, including allyl isothiocyanate (AITC; also known as mustard oil), allicin, components of tear gas, and air pollutant^3–7^. Because of its restricted expression pattern and unique function in irritant-sensing, it has long been proposed that blockade of TRPA1 could be a good therapeutic strategy to alleviate pain, itch, irritation, and asthma^8^. Indeed, several laboratories generated transgenic mouse lines that harbor null mutations in the Trpa1 gene (TRPA1 knockout (KO) mice) in order to study its function and to assess its validity as a target for various indications. Data generated using these mice supported the involvement of TRPA1 in cold sensation, inflammatory pain, chemotherapy-induced neuropathic pain, chronic itch, and asthma^9–15^.

In the course of our investigation into TRPA1’s value as a drug target for human chronic pain patients, we decided to generate a TRPA1 KO rat to enable a more detailed assessment of the role of TRPA1 channels in these indications. In particular, a rat model would enable more sophisticated *in-vivo* modeling than is possible in the mouse. Thus, we generated a TRPA1 KO rat in the Sprague Dawley strain using CRISPR and undertook a comprehensive investigation of the behavioral and physiological phenotype with particular interest in those areas where the contribution of TRPA1 had been highlighted by KO mouse studies. As expected, we found that the response to paw injection of mustard oil (AITC), a known potent activator of TRPA1, was completely absent in TRPA1 KO rats. However, no deficits were found in other models of nociceptive pain including radiant heat, von Frey, mechanical pinch (Randal-Sellito), cold sensitivity, and capsaicin injection. Furthermore, standard models of inflammatory and neuropathic pain were found to be normal, including complete Freunds Adjuvant (CFA) injection, strepotozotocin (STZ)-induced diabetic neuropathy, chemotherapy (Bortezomib)-induced painful neuropathy, and peripheral nerve injury-induced neuropathic pain (CCI model). On the other hand, the infiltration of immune cells into the lung in the rat OVA model of asthma was found to be dependent on TRPA1, supporting the potential use of TRPA1 inhibitors as a therapy for asthma.

## Results

### Generation and validation of the TRPA1 KO rat

**Figure 1:**
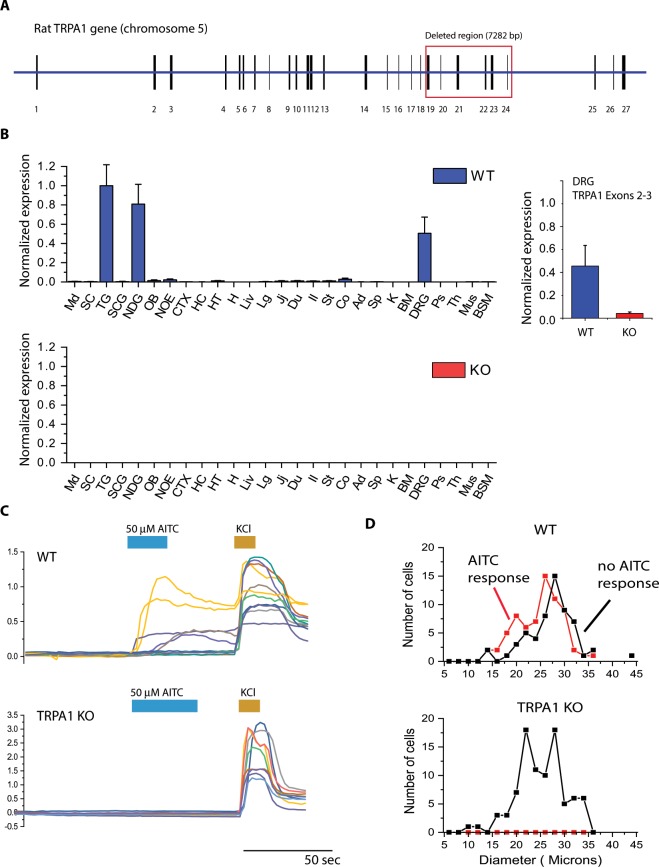
Knockout rats lack detectable full-length *trpa1* mRNA and cellular functional responses to TRPA1 agonists. (**A**) CRISPR technology was used to generate a 7282 base-pair deletion within the *Trpa1* gene in Sprague-Dawley rats, resulting to the removal of exons 19 through 24, which encode the full membrane-spanning portion of the TRPA1 ion channel protein. (**B**) Expression profile of *trpa1* mRNA across a panel of tissues from WT and TRPA1 KO rats. Tissues examined were medulla (Md), spinal cord (SC), trigeminal ganglia (TG), superior cervical ganglia (SCG), nodose ganglia (NDG), olfactory bulb (OB), nasal olfactory epithelium (NOE), cerebral cortex (CTX), hippocampus (HC), hypothalamus (HT), heart (H), liver (Liv), lung (Lg), jejunum (Jj), duodenum (Du), ileum (Il), stomach (St), colon (Co), adrenal gland (Ad), spleen (Sp), kidney (K), bone marrow (BM), dorsal root ganglia (DRG), pancreas (Ps), thymus (Th), quadriceps muscle (Mus), bronchial smooth muscle (BSM). On the right, inset, expression analysis of the non-deleted 5′ part of the TRPA1 transcript (exon 2–3 boundary) shows approximately 90% reduction in expression of the truncated transcript relative to the WT transcript. (**C**) Functional calcium imaging responses to the TRPA1 agonist AITC were observed in DRG neurons cultured from WT but not TRPA1 KO rats. All DRG neurons responded to a high KCl solution (30 mM KCl) in both WT and KO rats. (**D**) Size distributions of AITC-responsive and AITC non-responsive DRG neurons in WT and TRPA1 KO rats. Non-responsive neurons showed a similar size distribution between WT and KO DRG neurons, while no AITC-responsive neurons were observed in the KO rat. Overall, 128 WT neurons and 91 KO neurons were assessed to establish the size distributions.

In order to understand what therapeutic indications could potentially be affected by reducing TRPA1 function, we used CRISPR technology to generate a line of transgenic rats that lack TRPA1. These rats harbor a mutation that consists of a 7282 base-pair deletion in the Trpa1 gene that completely removes the membrane-spanning portion of the TRPA1 amino acid sequence (Fig. 1A). To verify that this mutation results in a non-functional allele, we performed qPCR experiments across a broad panel of tissues from rats homozygous for the mutated allele (TRPA1 KO rats) or littermates homozygous for the wildtype allele (WT rats). In WT rats, *trpa1* mRNA was found to be expressed at high levels in tissues containing sensory neurons, such as the dorsal root ganglia (DRGs), the trigeminal ganglia (TGs), and the nodose ganglia (NDGs). Lower amounts of *trpa1* mRNA were detected in gastrointestinal tissues, such as the stomach, colon, and small intestine, as well as the olfactory epithelium, olfactory bulb, and hypothalamus (Fig. 1B). No qPCR signal could be detected using probes within the 7282 bp deletion region as expected (Fig. 1B). We also examined the non-deleted part of the *trpa1* mRNA using a qPCR probe directed at the exon2–3 boundary, which is outside the deletion region, and found that the truncated mRNA was still present in the DRG, but about 10-fold lower in concentration (Fig. 1B, to the right). Because the pore region of the TRPA1 channel has been removed, the channel function of TRPA1 will be eliminated, but low amounts of a truncated transcript is still expressed.

We then conducted calcium imaging experiments to demonstrate that functional TRPA1 channels were no longer present in DRG neurons cultured from the TRPA1 KO rat. Briefly, we cultured rat DRG neurons from WT and KO rats and performed calcium imaging experiments using fura-2 ratiometric imaging and 50μM allyl isothiocyanate (AITC) as a TRPA1 agonist (Fig. 1C). In WT rats, AITC responses were observed in approximately 30–50% of DRG neurons immediately following the addition of AITC. We used 30 mM KCl to verify that the cells we were recording from were neurons. Side-by-side experiments with DRG neurons cultured from TRPA1 KO rats revealed no detectable AITC responses despite normal 30 mM KCl responses. This constitutes evidence that we have successfully generated KO rats in which TRPA1 expression and function are completely abolished. The size range of DRG neurons responding and not responding to AITC was measured and is similar to what has been reported in the literature^1,6,9,11,16–18^ (Fig. 1D).

### Examination of TRPA1 KO rats in models of nociception, acute pain, and itch

**Figure 2:**
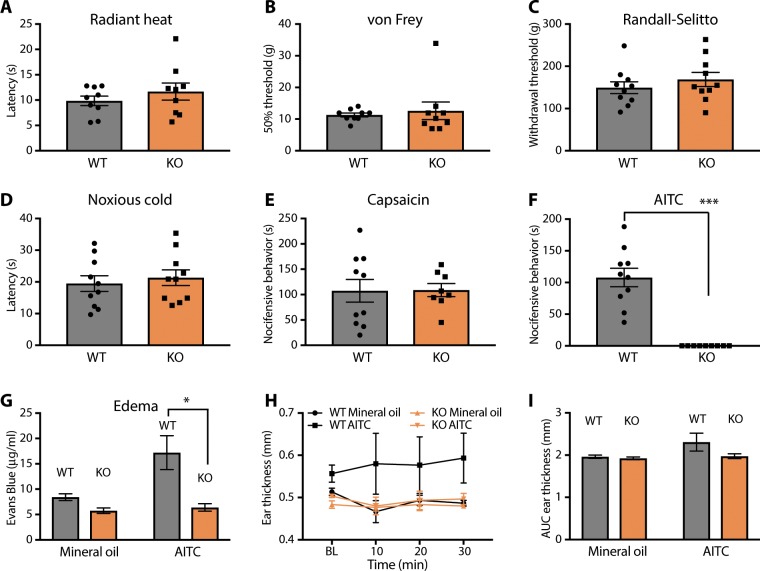
Behavioral and physiological responses to a TRPA1 agonist are absent in KO rats, yet acute nociception is intact. (**A–D**) TRPA1 KO rats are indistinguishable from WT littermates in their responses to hindpaw application of radiant heat (A), von Frey filaments (**B**), pinch (Randall-Selitto test) (**C**), and cold stimulation (**D**). (**E–F**) TRPA1 KO rats display flinching and hindpaw-directed licking and biting (i.e. nocifensive behavior) upon intraplantar injection of capsaicin (**E**), yet these behaviors are completely absent when AITC is injected (**F**). (**G–I**) Physiological inflammatory responses to AITC applied topically to the ear, including plasma extravasation (**G**) and edema (H, timecourse; I, area under the curve) are abrogated to the level of vehicle treatment in TRPA1 KO rats. *p < 0.05, ***p < 0.001. Error bars indicate SEM.

We performed a detailed neurological and histopathologic examination of TRPA1 KO rats and found them to be grossly normal and indistinguishable from WT littermates. Because TRPA1 has been implicated in pain and itch sensation, we focused our behavioral studies on investigating these sensory modalities in detail. In response to radiant heat (Fig. 2A) or von Frey filaments (Fig. 2B) applied to the hindpaw plantar surface, TRPA1 KO rats had normal withdrawal thresholds. They also responded normally to pressure applied to the hindpaw in the Randall-Selitto test (Fig. 2C).

Because TRPA1 KO mice have been variably reported to have normal^9^ or altered^11^ responses to noxious cold stimuli, we were especially interested in determining the behavior of our TRPA1 KO rats in the cold plantar assay^19^. Indeed, WT and TRPA1 KO rats had identical response latencies indicating no deficit in sensing noxious cold in the absence of TRPA1 (Fig. 2D).

Next, we injected the TRPV1 agonist capsaicin and measured flinching and licking directed toward the injected hindpaw. Both genotypes of rats responded similarly (Fig. 2E). In an analogous experiment, we performed intraplantar injection of the TRPA1 agonist AITC. In this case, nocifensive behavior was completely absent in TRPA1 KO rats (Fig. 2F). Furthermore, topical application of AITC to the ear of anesthetized animals elicited plasma extravasation only in WT and not TRPA1 KO rats (Fig. 2G). Measurements of ear thickness following AITC application trended toward showing a defect in TRPA1 KO rats, but failed to reach statistical significance. We interpret these findings to indicate that physiological and behavioral responses to direct TRPA1 activation are absent, yet acute nociception to other stimulus modalities are normal in TRPA1 KO rats.

**Figure 3:**
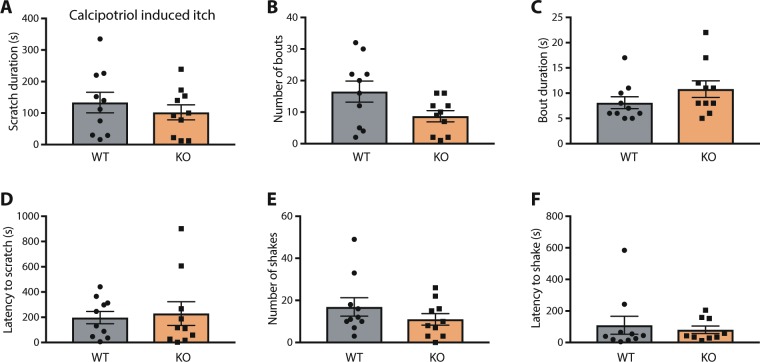
Scratching behavior is normal in TRPA1 KO rats in a chronic itch model. In the calcipotriol model of chronic itch/atopic dermatitis, TRPA1 KO rats were indistinguishable from WT littermates in several behavioral measures, including (**A**) time spent scratching, (**B**) number of scratching bouts, (**C**) average scratching bout duration, and (**D**) latency to begin scratching. Wet-dog shakes were also observed in this model, yet neither (**E**) number of shakes nor (**F**) latency to first shake were different between WT and TRPA1 KO rats. Error bars indicate SEM.

Using a model of chronic itch that involves topical application of the vitamin D analogue calcipotriol, we investigated scratching behavior in WT and TRPA1 KO rats. This model has previously been shown to induce spontaneous scratching in mice that is partially dependent on TRPA1 expression in mice^12^. We applied the calcipotriol solution to the nape once daily for seven days and recorded scratching and related behavior on day 12 after the beginning of the experiment. Indeed, we found that this treatment resulted in robust scratching in WT rats; however, it produced a similar amount of scratching in TRPA1 KO rats. This was the case whether we accounted for total time spent scratching, number of scratch bouts, average duration of individual scratch bouts, or latency to first scratch in a 30-minute observation period (Fig. 3A–D).

In addition to scratching, we noted that calcipotriol-treated rats engage in ‘wet dog shakes’. Systemic administration of icilin, an agonist of TRPA1^2,20,21^ and TRPM8^22,23^, has also previously been reported to induce wet dog shakes in rodents^24,25^, although TRPA1′s contribution to this behavior is unclear^26^. To test whether calcipotriol-induced wet dog shakes could be TRPA1-dependent, we also quantified these behaviors. Again, we observed no difference between WT and TRPA1 KO rats in either the number of wet dog shakes or the latency to the first shake during the observation period (Fig. 3E,F). Overall, our results do not support a robust contribution of TRPA1 to scratching or related behaviors in this rat model of chronic itch.

### Examination of TRPA1 KO rats in chronic pain models

**Figure 4:**
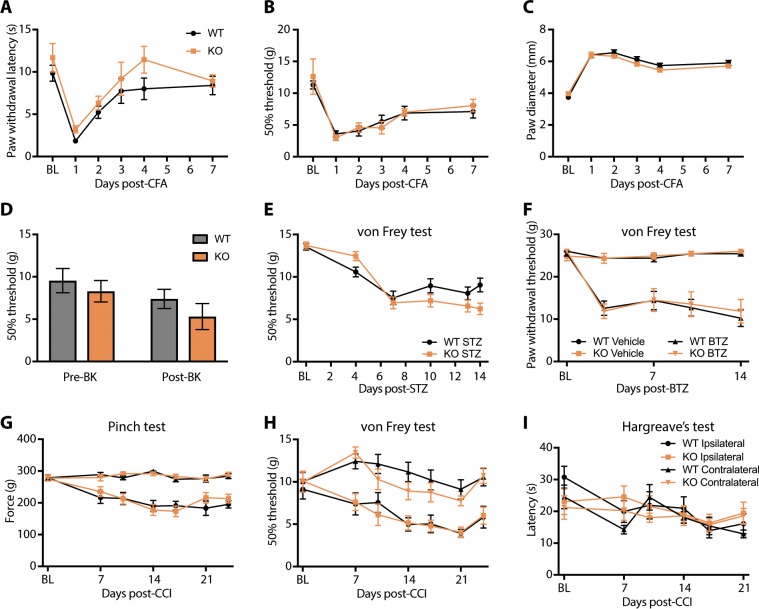
TRPA1 KO rats are similar to WT rats in several models of chronic pain. (**A–C**) Complete Freund’s adjuvant (CFA) model of inflammatory pain. Robust hypersensitivity to (**A**) radiant heat and (**B**) von Frey filaments, as well as (**C**) edema, developed similarly in both genotypes of rats. (**D**) A mild mechanical hypersensitivity developed within 2 h of intraplantar injection of bradykinin that was similar in both genotypes. (**E**) In the streptozotocin model of Type I painful diabetic neuropathy, both WT and TRPA1 KO rats developed mechanical hypersensitivity with a similar magnitude and timecourse. (**F**) In the bortezomib model of chemotherapy-induced painful neuropathy, both WT and TRPA1 KO rats developed mechanical hypersensitivity to a similar degree. (**G–I**) Chronic constriction injury (CCI) model of neuropathic pain. (**G**) Mechanical hyperalgesia (Randall-Selitto test) and (**H**) mechanical allodynia (von Frey filaments) developed in both genotypes to a similar extent on the lesioned side, and (**I**) cold allodynia was not robustly observed in either genotype. Error bars indicate SEM.

Next, we turned to models of chronic pain, as protection has been reported in TRPA1 KO mice in a number of similar experimental paradigms^9,11,13,14,27^. In the CFA model of chronic inflammatory pain, strong hypersensitivity to both heat and mechanical stimuli, as well as paw edema, were observed that mostly resolved over the course of a week. These effects were indistinguishable between WT and TRPA1 KO rats (Fig. 4A–C).

It is possible that inflammation in the CFA model is strong enough to mask a partial contribution of TRPA1 to inflammatory pain, so we next used a bradykinin model that produces shorter-lasting inflammation and milder sensitization, previously shown to be largely reduced in TRPA1 KO mice^9,11^. Indeed we observed only a mild sensitization to mechanical stimuli at 2 h post-injection, which was similar in both genotypes of rats (Fig. 4D). Under these conditions, heat hypersensitivity did not develop in either WT or TRPA1 KO rats (data not shown).

Painful diabetic neuropathy has been linked to TRPA1 in animal studies using antagonists^28,29^, but to our knowledge data have not yet been reported in knockout animals. Systemic administration of the pancreatic beta cell toxin streptozotocin (STZ) induces a model of Type I diabetes showing increased sensitivity to mechanical stimuli. When we administered STZ to WT and TRPA1 KO rats, we found that it induced hyperglycemia and mechanical allodynia that developed over the course of several days; both genotypes were affected to a similar degree (Fig. 4E).

Chemotherapy-induced neuropathic pain is another potential indication of interest where TRPA1 deletion has been described to provide protective benefit in mice^13,14,27^. When we administered the proteasome inhibitor and chemotherapeutic agent bortezomib (BTZ) systemically to WT and TRPA1 KO rats, it induced hypersensitivity to mechanical stimuli applied to the hindpaw that was indistinguishable between genotypes (Fig. 4F). Vehicle-treated control animals were unaffected in this outcome measure.

Finally, we applied the chronic constriction injury (CCI) model of neuropathic pain to the sciatic nerve^30^. Using two different measures of mechanical threshold testing (digital Randall-Selitto, Fig. 4G; and von Frey filaments, Fig. 4H), we observed significant induction of mechanical hyperalgesia and allodynia ipsilateral to the constricted nerve. The onset, duration, and magnitude of these effects was indistinguishable between WT and TRPA1 KO rats. Withdrawal thresholds in both these tests could be reversed to baseline levels by administering gabapentin or duloxetine, with no differences in drug effect between genotypes (data not shown). We additionally tested cold sensitivity in the CCI model using the cold plantar assay. No significant cold allodynia was detected for either genotype at any timepoint post-CCI surgery under the present experimental conditions (Fig. 4I).

### Examination of TRPA1 KO rats in the OVA model of asthma

**Figure 5:**
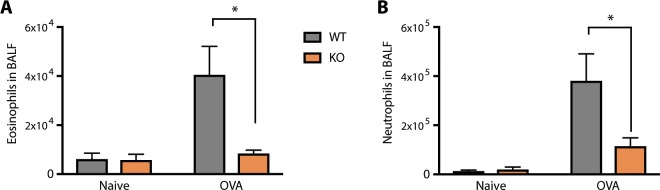
Robust protection by TRPA1 deletion in the ovalbumin sensitization model of asthma. WT and TRPA1 KO Rats were sensitized or not (naïve group) and challenged with OVA. 24 hours after the final OVA challenge (28 days after initial OVA sensitization), bronchial lavage was analyzed for eosinophils and neutrophils by FACS. (**A**) Eosinophils increased 7-fold in the BALF in WT rats but not in TRPA1 KO rats (Tukey’s multiple comparisons test; p = 0.0383). (**B**) Neutrophils increase 29-fold in the BALF in WT rats but not TRPA1 KO rats (Tukey’s multiple comparisons test; p = 0.0202). *p < 0.05. Error bars indicate SEM.

Apart from pain and itch, TRPA1 has also been proposed to contribute critically to airway inflammation in asthma. In particular, it has been shown in the ovalbumin (OVA) model of asthma that TRPA1 KO mice have a decreased inflammatory response as measured by reduced numbers of infiltrated leukocytes in the bronchoalveolar lavage fluid (BALF) after OVA challenge^10^. We decided to test whether this was also true for TRPA1 KO rats. After establishing OVA sensitization, rats were exposed to 2% OVA as an aerosol for 30 min on each of four consecutive days, and BALF was collected 24 h after the last exposure. We observed a strong increase in infiltrated leukocytes in WT OVA-challenged rats compared to naïve rats that was clearly dampened in TRPA1 KO OVA-challenged rats. In particular, eosinophils (Fig. 5A) and neutrophils (Fig. 5B) were counted in lower numbers in TRPA1 KO than WT BALF after OVA challenge. Thus, we provide genetic evidence in a second species for the functional contribution of TRPA1 to airway inflammation in an asthma model.

## Discussion

Here we describe a thorough characterization of the physiological and behavioral phenotype of rats harboring a null allele of *Trpa1* with respect to models of pain, itch, and asthma. This rat was constructed by removal of a 7282 base-pair region that includes the transmembrane part of the TRPA1 channel using CRISPR technology. While this eliminates ion channel function, a truncated transcript that encodes the N-terminal ankyrin repeat region was still present, though at reduced levels. We found rats lacking TRPA1 to be deficient in mounting behavioral and inflammatory responses to the TRPA1 agonist AITC, and they recruit fewer immune cells to the airway in response to asthmatic challenge compared to WT rats. Apart from these key differences, TRPA1 KO rats were indistinguishable from their WT littermates in all other assays we examined, including tests of neurological function and acute nociception, as well as numerous models of chronic pain and itch.

When examining a germline knockout mouse or rat, developmental compensation should be considered as a potential explanation for the KO having no phenotype despite observations of pharmacological efficacy of inhibitors. For example, several labs have demonstrated efficacy of TRPA1 inhibitors in the CFA model of inflammatory pain^31–34^. In fact in one case, direct evidence for developmental compensation was observed since the CFA model was found to be normal in the TRPA1 KO mouse, but a TRPA1 inhibitor was able to block CFA-induced mechanical hyperalgesia in the WT mouse but not the TRPA1 KO mouse^34^. However, these experiments used low potency first-generation TRPA1 inhibitors such as HC030031 and AP18, increasing the likelihood that some of the effects of these compounds could be off-target. Indeed, when high potency TRPA1 inhibitors have been tested in the CFA model (and neuropathic pain models), only limited, if any, efficacy has been observed^35^.

It was surprising that TRPA1 KO rats display normal scratching behavior in the calcipotriol-induced chronic itch model, since several high-quality studies showed a robust requirement for TRPA1 in itch using KO mice^12,15,36^. One possible explanation for this discrepancy is that the studies were done in different species: TRPA1 may be involved in itch signaling in mice but not in rats. Of note, however, it was recently reported that scratching responses to intradermal injection of the pruritogen chloroquine are indistinguishable between WT and TRPA1 KO mice^37^. It may be that TRPA1 contributes significantly to itch under a restricted set of conditions that reflects an interplay of strain, species, and laboratory environment.

Whether TRPA1 contributes to behavioral responses to cold stimuli has been a subject of controversy. Diminished sensitivity to cold was reported in one strain of TRPA1 KO mice^11^, yet responses to cold were reported to be normal in another^9^. Our results with TRPA1 KO rats indicate that TRPA1 does not play a major role in responses to cold, at least under the conditions use in our experiments. In general, while mouse and rat TRPA1 channels can be activated similarly by cold stimuli *in vitro*^38^, their genetic deletion in either species fails to lead to a dramatic effect on behavioral responses to noxious cold stimuli.

A body of literature exists on the potential for functional interaction between TRPA1 and TRPV1^39–44^. Extrapolation of these *in vitro* findings might predict altered TRPV1 function in TRPA1 KO animals. In our experiments, noxious heat stimulation or intraplantar injection of the TRPV1 agonist capsaicin produced exactly the same effects in rats expressing or lacking TRPA1, indicating minimal functional impact on TRPV1 at the behavioral level.

One striking difference between WT and TRPA1 KO rats is in their response to the TRPA1 agonist AITC. In the absence of TRPA1, nocifensive and inflammatory (plasma extravasation, edema) reactions to AITC were eliminated. This reinforces the now well-established view that TRPA1 is the only receptor for AITC in the nociceptive and inflammatory systems. Anecdotally, we noted that TRPA1 KO rats still maintain avoidance behaviors upon presentation with an AITC-infused cotton swab, indicating that there may be further receptor(s) for this chemical, possibly in the olfactory system. Regardless, if our results in rats translate to humans, non-invasive measurement of blood flow changes after topical application of AITC could be used as a clinical biomarker of target engagement by TRPA1 inhibitors^28^.

TRPA1 KO rats were largely protected from immune cell infiltration into bronchoalveolar lung fluid in the OVA model of asthma. Thus, our findings recapitulate previous OVA model studies in TRPA1 KO mice^10^. Thus, TRPA1 appears to make a robust, cross-species contribution to airway inflammation in preclinical asthma models. Inhaled irritants likely activate TRPA1 expressed in sensory neurons that innervate the airways, which leads to peripheral release of vasoactive and inflammatory substances that influence the infiltration of eosinophils and neutrophils to the lungs. Based on our findings and those of others using genetic deletion, we suggest that pharmacological blockade of TRPA1 could provide therapeutic benefit in human respiratory disorders.

Overall, our study provides a thorough phenotypic mapping of pain and asthma phenotype of the TRPA1 KO rat. We found that previous reports of protection from airway inflammation in TRPA1 KO mice were reproducible in a second species using our novel TRPA1 KO model. However, comprehensive behavioral phenotyping revealed no difference between rats expressing or lacking TRPA1 in acute nociception or several models of chronic pain and chronic itch with the exception of nociception following direct activation of TRPA1. We acknowledge that compensatory mechanisms during development might have occurred in the TRPA1 KO rat that would prevent us from observing TRPA1-dependent pain phenotypes, which should be examined further. It is our hope that this new TRPA1 KO rat model can be used as a tool to further examine the role that TRPA1 plays in pain and itch and as a control to demonstrate that observed effects of TRPA1 antagonists in the rat are really working through the TRPA1 target.

## Materials and Methods

### Animals

Experimental procedures involving animals were approved by Genentech’s Institutional Animal Care and Use Committee and conducted in accordance with the recommendations of the International Association for the Study of Pain. Rats at least 8 weeks of age of both sexes were used. All behavioral studies were performed by experimenters blinded to genotype. Statistical analysis of behavioral studies is presenting as a statistical table (Supp. Table 1).

### Generation of TRPA1 knockout rats

Rats harboring a 7282 bp deletion spanning *Trpa1* exons 19 through 24, corresponding to genomic position RGSC 6.0/rn6 chr5:3,818,620-3,825,901, were obtained by cytoplasmic co-injection of Cas9 mRNA and sgRNAs into Sprague-Dawley zygotes using established methods, and the resulting mosaic founders were analyzed for editing at the top 5 algorithm-predicted off-targets, as previously described (*Anderson et al., Nat.Meth. 2018*). Mosaic founders without off-targets were bred to wildtype Sprague-Dawley mates to generate F1 heterozygous progeny for subsequent intercrossing. The sgRNA sequences used to target *Trpa1* exons 19 and 24, respectively, are ex19_gRNA2 5′-gAGAGCTCATATGATGAACCT-3′ and ex24_gRNA2 5′-gGGCAGTTGGGGACATTGCTG-3′ (5′ mismatch indicated by lowercase g).

### RT-PCR

To examine TRPA1 tissue distribution, multiple tissues, including DRGs, were collected from WT and TRPA1 KO rats. Dissected tissues were stored in RNAlater solution (Invitrogen, AM7021) at −20 °C until required for analysis. Total RNA was isolated using the RNeasy Plus-96 kit (QIAGEN, #74804) and further reverse transcribed into complementary DNA using a TaqMan gene expression kit (Ambion, Cells-to-CT kit, AM1728). qRT-PCR assays were performed in an ABI viiA7 system (Applied Biosystems). Assay primers and probes for TRPA1 were designed by us to detect the deleted regions of the TRPA1 gene, and synthesized by IDTdna. The experiment included three assays with probes designed to span exons 20–21, 21–22 and 22–23 of the TRPA1 gene and yielded very similar data (only the 21–22 data is shown in Fig. 1B). All three assays were run in duplicate with β-actin as an internal control. Studies of transcript expression outside of the deleted region was done using a probe for the exon 2–3 boundary (Rn01473803, Applied Biosystems, ThermoFisher). Rat β-actin assay primers and probes were obtained from Applied Biosystems. Threshold cycle (C_t_) data for TRPA1 and β-actin were obtained using the Viia7 software and analyzed using Origin (OriginLab corporation). Relative expression was calculated by averaging data from the three assays and normalizing relative to TRPA1 expression in the trigeminal ganglion.

### OVA model

Ovalbumin (OVA), from chicken egg white, was purchased from Sigma (catalog number A5503-5G, lot SLBK1399V). Alum was purchased from Pierce (catalog number 777161, lot 1B11707). Rats were immunized on day 0 with intraperitoneal administration of 150 µg ovalbumin mixed with 40 mg of alum diluted in sterile PBS. 28 days after sensitization, rats were challenged with 2% OVA in PBS aerosolized via a nebulizer for 30 minutes for four consecutive days. 24 hours after the final treatment, rats were euthanized by CO_2_ inhalation. Post-euthanasia, BAL fluid was collected for total and differential cell counts. There were 8 animals per group.

### Analysis of Bronchoalveolar lavage (BAL) fluid

Lungs were lavaged with PBS. Cell counts were determined by FACS analysis using a known quantity of Fluoresbrite YG microspheres from Polysciences, Inc. as a spike-in standard. Red blood cells from BAL were lysed with 1.5 mL of ACK lysing buffer and washed with FACS buffer. The percentage of Eosinophils (Sytox−, CD45+, CD11b+, RP-1−) and Neutrophils (Sytox−, CD45+, CD11b+, RP-1+) cells were determined by FACS analysis and total cell numbers were calculated.

### DRG acute culture

Rats were euthanized by CO_2_ inhalation and dorsal root ganglia (DRG) were isolated bilaterally. The ganglia were first incubated at 37 °C and 5% CO_2_ with 1 mg/ml collagenase Type IV (Sigma C1889) for 40 min, followed by incubation with 0.05% trypsin (Sigma T9935) for 45 min. Ganglia were washed and then dissociated into single somata via trituration through a P200 pipette tip. Neurons were filtered carefully, under sterile conditions, through a 70 micron cell filter and plated onto Poly-D-Lysine-coated glass coverslips placed in a multiwell culture dish. These coverslips were incubated for 1 h at 37 °C and 5% CO_2_ to allow adherence. Coverslips were then flooded with complete cell medium consisting of DMEM, 10% heat-inactivated horse serum, 2 mM l-glutamine, 0.8% d-glucose, 100 units penicillin, and 100 mg/ml streptomycin.

### Calcium imaging

Calcium imaging experiments were conducted 15–24 hours after plating. Cells were loaded with Fura-2AM ester (Molecular Probes) and placed in a perfusion chamber. Ratiometric fluorescence measurements were made by measuring emission to sequential excitation at 340 and 380 nM, using a DG-4 (Sutter Instrument Co.) wavelength switching system. Fluorescence data points were collected at 0.5 Hz using AxioVision software (Zeiss) and analyzed using Origin (OriginLab Corporation). During the recoding cells were continuously perfused with normal saline (155 mM NaCl, 2 mM KCl, 1 mM MgCl_2_, 1.8 mM CaCl_2_, 10 mM HEPES- pH 7.4, 5 mM Glucose). TRPA1 activity was measured by perfusing cells with saline containing 50 μM AITC (Sigma). Neurons in the field of observation were identified by their Calcium influx response to 50 mM KCl-containing saline. Only neurons were included in the analysis. Responses to AITC were normalized to mean KCl response for each experiment.

### Behavioral testing

All studies were performed by experimenters blinded to genotype.

### Hindpaw radiant heat (Hargreaves’s) test

Rats were acclimated for 15–30 min in individual Plexiglas test chambers in which they could move freely, on a room-temperature glass platform. A radiant heat source was focused on the plantar surface of one hindpaw of the rat, and latency to voluntarily withdraw the paw was measured. Three trials were performed, spaced at least 5 min apart to avoid sensitizing the paws. The intensity of the radiant heat source was set to result in a withdrawal latency of about 10 s in healthy control animals. A maximum cutoff latency of 30 seconds was used to avoid heat damage to the paws. *von Frey test of mechanical threshold*. Rats were habituated for 15–30 min in individual Pexiglas test chambers on an elevated wire mesh surface. Nylon filaments that have been calibrated to deliver precise forces were applied one at a time to the plantar surface of one hindpaw of each rat, following the up-down method^45^. Briefly, if the rat withdrew its hindpaw in response to stimulation with a filament, it was stimulated again later with the next weaker filament in the series; if the rat did not react to a given filament, it was stimulated again with the next stronger filament in the series. Stimulation continued until six responses were recorded surrounding the withdrawal threshold. Stimuli presented to rats ranged from 0.4 g to 26 g. *Randall-Selitto test*. Rats were acclimated to handling by experimenters. To perform the test, rats were lightly restrained by the experimenter and one hindpaw of the rat was positioned on a flat platform and a roundly tapered plinth was laid on top of the paw. The Randall-Selitto apparatus (Analgesymeter, Ugo Basile) was activated and a smooth force ramp was applied at the point of contact between the plinth and the paw. When the rat struggled, attempted to withdraw its paw, or vocalized the trial was stopped and the amount of force that was delivered was recorded. The starting pressure was 0 g, the rate of increase was 32 g/s, and the maximal cutoff was 200 g. Three to five trials were performed per animal, spaced at least 5 min apart.

### Cold plantar test

Rats were acclimated for 15–30 min in individual Plexiglas test chambers on a room-temperature glass platform. Dry ice was crushed and loaded into a 3 ml plastic syringe and compressed to form a pellet with flat surface. The dry ice pellet was then extended out of the syringe and pressed firmly against the glass beneath one hindpaw of the rat. The time to withdraw the hindpaw was recorded, with a cutoff of 30 seconds to avoid injury. Method adapted from Brenner *et al*.^19^.

### Calcipotriol-induced Itch

Rats were briefly anesthetized under isoflourane inhalation to effect, and a 3 cm by 3 cm patch of fur on the back, just below the nape of the neck, was shaved with clippers. Once a day for 7 days, 100 µl of 200 µM calcipotriol (Sigma, in 100% ethanol) was applied topically to the shaved area with a pipet. On the twelfth day following first application, rats were placed in individual Plexiglas chambers on a glass platform and allowed to acclimate for 10 minutes. Rats were videotaped for 30 minutes and then returned to their home-cage. Videos were scored for itch-related behaviors later offline. Based on methods from Morita *et al*., 2015. *Intraplantar capsaicin*. Rats were acclimated to handling by experimenter prior to the experiment. Each rat was lightly restrained in a towel, and an intraplantar injection of 20 μl of a solution containing 3 μg capsaicin (vehicle: 10% ethanol and 0.5% Tween 80 in saline) was made to one hindpaw using a 30 gauge disposable needle attached to a luer-tipped Hamilton syringe. The rat was then immediately placed in a Plexiglas test chamber on room temperature glass with mirrors arranged below to allow observation from both side and bottom views. The responses of up to 6 rats at a time were videotaped for later scoring of nocifensive behaviors. Paw diameter was measured immediately before injection and at the end of the test using a digital thickness gauge (Mitutoyo).

### Intraplantar AITC

Procedure as described for intraplantar capsaicin, instead with injection of 25 microliters of 0.1% AITC solution diluted in mineral oil.

### CFA model

An intraplantar injection of 10 µl of an emulsion of equal parts CFA and mineral oil was made to one hindpaw. Behavioral testing was performed on days 1, 2, 3, 4, and 7 following injection. Paw thickness was measured after behavioral testing using a digital thickness gauge (Mitutoyo).

### Bradykinin model

Rats were injected intraplantar with 25 µl of bradykinin (100 ng in sterile saline). Behavioral testing was performed prior to injection for baseline measurements, and at 30 minutes post-injection (Hargreaves’s) and 2 hours post-injection (von Frey).

### Chronic constriction injury (CCI) model

The chronic constriction injury surgery was used to induce neuropathic pain in rats^30^. Prior to and on days 7, 10, 14, 17, and 21 post-surgery, animals were assessed for mechanical allodynia, mechanical hyperalgesia, and cold allodynia. Mechanical allodynia was assessed using von Frey filaments according to the “up-down” method^45^. Mechanical hyperalgesia was assessed by applying a pressure stimulus using a Randall-Sellito apparatus (IITC) to the plantar surface of the hind paw gradually until the first observed nocifensive behavior (vocalization, struggle, or withdrawal). One reading per paw was taken and a maximum stimulus cutoff of 300 grams was used to prevent injury to the animal. Cold allodynia was measured by applying an ~15 mm wide pellet of dry ice to a glass platform underneath the plantar surface of the hind paw until nocifensive paw withdrawal occurred or until the maximum time of 90 seconds was reached. Each paw was tested two times per time point, with a minimum of 5 minutes between each nocifensive response.

### Streptozotocin (STZ) model

Mechanical allodynia was assessed prior to STZ (Sigma) administration and was measured using von Frey filaments according to the up-down method^45^. Animals were then administered 50 mg/kg STZ intravenously on Day 0. On Day 4, animals were assessed for blood glucose levels and mechanical allodynia. Animals with blood glucose levels below 300 mg/dL were excluded from the study. On Days 7 and 10, animals were assessed for mechanical allodynia. On Day 13, animals were assessed for mechanical allodynia and sorted into groups based on the Day 13 baseline values. Animals were administered gabapentin (100 mg/kg; p.o. in saline) or water and mechanical allodynia was assessed 1, 2, and 4 hours post-dose. On Day 14, mechanical allodynia was assessed prior to duloxetine (30 mg/kg; p.o. in saline) or PBS administration and 1, 2, and 4 hours post-dose. Body weight was assessed weekly and blood glucose levels were re-assessed prior to humane euthanasia.

### Bortezomib (BTZ) model

Rats were tested for their paw withdrawal threshold (PWT) values for non-noxious mechanical sensitivity using von Frey filaments according to the up-down method^45^. The responses from both hind paws were averaged at each time point. Rats were injected with either BTZ (0.5 mg/kg; i.p. in 5% Tween-80, 5% ethanol in 90% saline) or vehicle on Day 0. Post BTZ PWT was assessed on Days 3, 7, 10, and 14. On Day 14 after baseline evaluation, all rats were administered Gabapentin by oral gavage and tested 1 hour post dose.

## Supplementary information

Supplementary Info.
