# Supplementary material for: Behavioral characterization of a CRISPR-generated TRPA1 knockout rat in models of pain, itch, and asthma
Source: Sci Rep. 2020 Jan 22;10:979. doi: 10.1038/s41598-020-57936-5 (PMC6976688; doi:10.1038/s41598-020-57936-5)
Supplement: Supplementary file 1 — Supplementary Info. [file 41598_2020_57936_MOESM1_ESM.docx]

**Behavioral characterization of a CRISPR-generated TRPA1 knockout rat in models of pain, itch, and asthma**

**Authors**

Rebecca M. Reese^1†^, Michelle Dourado^1†^, Keith Anderson^3^, Søren Warming^3^, Kimberly L. Stark^1^, Alessia Balestrini^2^, Eric Suto^2^, Wyne Lee^2^, Lorena Riol-Blanco^2^, Shannon D. Shields^1^, David H. Hackos^1^*

**Affiliations**

^1^Department of Neuroscience, Genentech, 1 DNA Way, South San Francisco, CA 94080 USA

^2^Department of Immunology, Genentech, 1 DNA Way, South San Francisco, CA 94080 USA

^3^Department of Molecular Biology, Genentech, 1 DNA Way, South San Francisco, CA 94080 USA

^†^These authors contributed equally to this work.

*To whom correspondence should be addressed: hackos.david@gene.com.

**Supplemental Table 1**

| Figure panel | Assay | Statistical test; findings | Post-hoc analysis (adjusted p-values) | Number of subjects |
| --- | --- | --- | --- | --- |
| 2A | Hargreaves test | Unpaired t test  WT vs. KO  p=0.3556 | n/a | n=9/genotype |
| 2B | von Frey test | Unpaired t test  WT vs. KO  p=0.6509 | n/a | n=9/genotype |
| 2C | Randall-Selitto test | Unpaired t test  WT vs. KO  p=0.3829 | n/a | n=10/genotype |
| 2D | Cold plantar assay | Unpaired t test  WT vs. KO  p=0.6058 | n/a | n=10/genotype |
| 2E | i.pl. capsaicin flinching | Unpaired t test  WT vs. KO  p=0.9611 | n/a | n=10 WT, 8 KO |
| 2F | i.pl. AITC flinching | Unpaired t test  WT vs. KO  p<0.0001 | n/a | n=10 WT, 9 KO |
| 2G | AITC ear plasma extravasation (Evans Blue) | Two-way ANOVA  Genotype: p=0.0493  Treatment: p=0.0116  Interaction: p=0.0786 | Sidak’s multiple comparisons test  WT:  AITC vs. mineral oil: p=0.0410  KO:  AITC vs. mineral oil: p=0.9672 | n=3 WT AITC, 3 KO AITC, 2 WT mineral oil, 3 KO mineral oil |
| 2H | AITC ear thickness (timecourse) | Two-way repeated measures ANOVA  Genotype: p=0.1335  Time: p=0.6251  Interaction: p=0.7305 | n/a | n=3/genotype |
| 2I | AITC ear thickness (AUC) | Two-way ANOVA  Genotype: p=0.1339  Treatment: p=0.1170  Interaction: p=0.2252 | n/a | n=3/genotype |
| 3A | Scratch duration | Unpaired t test  WT vs. KO  p=0.4496 | n/a | n=10/genotype |
| 3B | Scratch bouts | Unpaired t test  WT vs. KO  p=0.0547 | n/a | n=10/genotype |
| 3C | Bout duration | Unpaired t test  WT vs. KO  p=0.1999 | n/a | n=10/genotype |
| 3D | Latency to scratch | Unpaired t test  WT vs. KO  p=0.7647 | n/a | n=10/genotype |
| 3E | Number of shakes | Unpaired t test  WT vs. KO  p=0.2681 | n/a | n=10/genotype |
| 3F | Latency to shake | Unpaired t test  WT vs. KO  p=0.6603 | n/a | n=10/genotype |
| 4A | CFA Hargreaves test | Two-way repeated measures ANOVA  Genotype: p=0.1766  Time: p<0.0001  Interaction: p=0.7354 | n/a | n=9/genotype |
| 4B | CFA von Frey test | Two-way repeated measures ANOVA  Genotype: p=0.8117  Time: p<0.0001  Interaction: p=0.8448 | n/a | n=9/genotype |
| 4C | CFA paw diameter | Two-way repeated measures ANOVA  Genotype: p=0.3572  Time: p<0.0001  Interaction: p=0.1546 | n/a | n=9/genotype |
| 4D | BK von Frey test | Two-way repeated measures ANOVA  Genotype: p=0.2938  Time: p=0.0486  Interaction: p=0.7365 | n/a | n=8 WT, 10 KO |
| 4E | STZ von Frey test | Two-way repeated measures ANOVA  Genotype: p=0.1744  Time: p=0<0.0001  Interaction: p=0.0028 | n/a | n=25/genotype |
| 4F | BTZ von Frey test | Two-way repeated measures ANOVA  Vehicle:  Genotype: p>0.9999  Time: p=0.3557  Interaction: p=0.8389  BTZ:  Genotype: p=0.8293  Time: p<0.0001  Interaction: p=0.9403 | n/a | n=10 WT vehicle, 10 WT BTZ, 10 KO vehicle, 8 KO BTZ |
| 4G | CCI dRS | Two-way repeated measures ANOVA  Contralateral:  Genotype: p=0.7937  Time: p=0.1455  Interaction: p=0.6656  Ipsilateral:  Genotype: p=0.7425  Time: p<0.0001  Interaction: p=0.4014 | n/a | n=15/genotype |
| 4H | CCI von Frey test | Two-way repeated measures ANOVA  Contralateral:  Genotype: p=0.3327  Time: p=0.0002  Interaction: p=0.5206  Ipsilateral:  Genotype: p=0.9990  Time: p<0.0001  Interaction: p=0.9307 | n/a | n=15/genotype |
| 4I | CCI cold plantar assay | Two-way repeated measures ANOVA  Contralateral:  Genotype: p=0.8490  Time: p=0.0068  Interaction: p=0.0520  Ipsilateral:  Genotype: p=0.9724  Time: p=0063  Interaction: p=0.3961 | n/a | n=15/genotype |
| 5A | OVA model, BALF eosinophils | One-way ANOVA  p=0.0063 | Tukey's multiple comparisons test  WT:  Naïve vs. OVA: p=0.0383  KO:  Naïve vs. OVA: p=0.9959 | n=5 WT naÏve, 5 KO naïve, 10 WT OVA, 10 KO OVA |
| 5B | OVA model, BALF neutrophils | One-way ANOVA p=0.0060 | Tukey's multiple comparisons test  WT:  Naïve vs. OVA: p=0.0202  KO:  Naïve vs. OVA: p=0.8487 | n=5 WT naÏve, 5 KO naïve, 10 WT OVA, 10 KO OVA |
